# Supplementary material for: NGS-Based Genetic Analysis in a Cohort of Italian Patients with Suspected Inherited Myopathies and/or HyperCKemia
Source: Genes (Basel). 2023 Jul 2;14(7):1393. doi: 10.3390/genes14071393 (PMC10379733; doi:10.3390/genes14071393)
Supplement: Supplementary file 1 [file genes-14-01393-s001.zip › Fig S1.pdf]

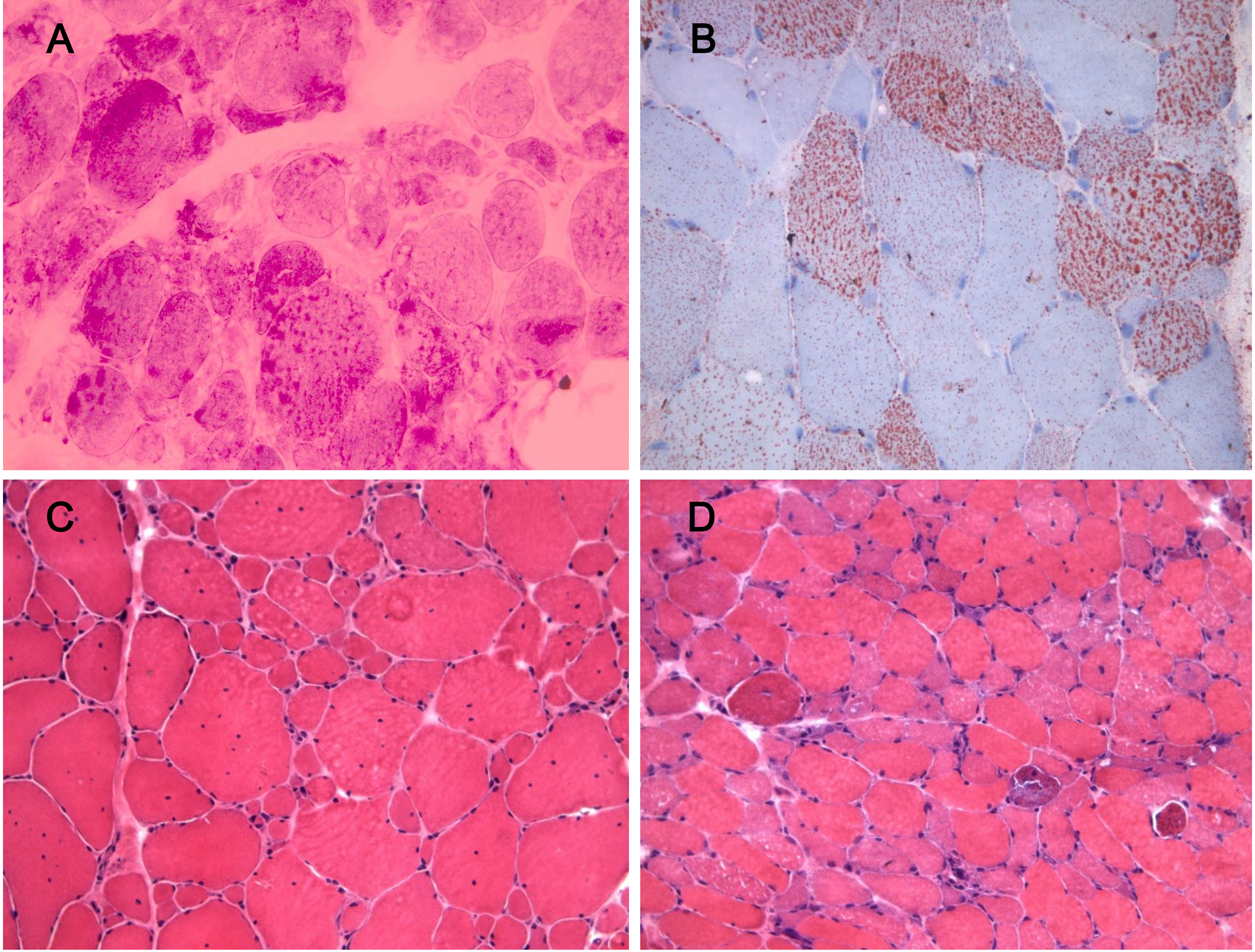

Supplementary Figure S1: Histological findings reveal (**A**) vacuolated fibers filled with periodic acid-schiff (PAS) positive material (glycogen), 20x ; (**B**) lipid storage in muscle fibers, Oil-red-O, 20x. Hematoxylin-eosin staining show a biphasic variation in muscle fiber size with clusters of rounded atrophic muscle fibers (**C**), and necrotic muscle fibers without any cellular infiltrates (**D**).
